# A Damaging *COL6A3* Variant Alters the *MIR31HG*‐Regulated Response of Chondrocytes in Neocartilage Organoids to Hyperphysiologic Mechanical Loading

**DOI:** 10.1002/advs.202400720

**Published:** 2024-07-17

**Authors:** Niek GC Bloks, Zainab Harissa, Giorgia Mazzini, Shaunak S Adkar, Amanda R Dicks, Ghazaleh Hajmousa, Nancy Steward, Roman I. Koning, Aat Mulder, Berend B.R. de Koning, Margreet Kloppenburg, Rodrigo Coutinho de Almeida, Yolande FM Ramos, Farshid Guilak, Ingrid Meulenbelt

**Affiliations:** ^1^ Leiden University Medical Center Leiden 2333 ZC The Netherlands; ^2^ Washington University Saint Louis MO 63110 USA; ^3^ Shriners Hospitals for Children Saint Louis MO 63110 USA

**Keywords:** collagen type VI, cyclooxygenase 2, exome sequencing, osteoarthritis

## Abstract

The pericellular matrix (PCM), with its hallmark proteins collagen type VI (COLVI) and fibronectin (FN), surrounds chondrocytes and is critical in transducing the biomechanical cues. To identify genetic variants that change protein function, exome sequencing is performed in a patient with symptomatic OA at multiple joint sites. A predicted damaging variant in *COL6A3* is identified and introduced by CRISPR‐Cas9 genome engineering in two established human induced pluripotent stem cell‐derived in‐vitro neocartilage organoid models. The downstream effects of the *COL6A3* variant on the chondrocyte phenotypic state are studied by a multi‐omics (mRNA and lncRNA) approach in interaction with hyper‐physiological mechanical loading conditions. The damaging variant in *COL6A3* results in significantly lower binding between the PCM proteins COLVI and FN and provokes an osteoarthritic chondrocyte state. By subsequently exposing the neocartilage organoids to hyperphysiological mechanical stress, it is demonstrated that the *COL6A3* variant in chondrocytes abolishes the characteristic inflammatory signaling response after mechanical loading with *PTGS2*, *PECAM1*, and *ADAMTS5*, as central genes. Finally, by integrating epigenetic regulation, the lncRNA *MIR31HG* is identified as key regulator of the characteristic inflammatory signaling response to mechanical loading.

## Graphical abstract

Osteoarthritis (OA) results from interaction of genetic susceptibility and environmental stressors, like mechanical stress. The pericellular matrix (PCM), featuring collagen type VI (COLVI) and fibronectin (FN), surrounds chondrocytes and is critical in transmitting biomechanical signals. Introducing a damaging *COL6A3* variant into induced pluripotent stem cell‐derived neocartilage models promotes an osteoarthritic chondrocyte phenotype and impairs PCM protein binding. This leads to an impaired repair response, regulated by *MIR31HG*.

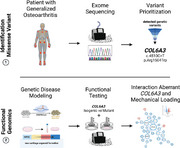

## 1 Introduction

Osteoarthritis (OA) is a complex, multifactorial disease revolving around the interplay between genetic and environmental risk factors. In particular, biomechanical cues play a critical role in not only joint health but also drive the onset and progression of disease.^[^ ^1^ ^]^ Indeed, physiologic levels of mechanical loading by virtue of physical exercise can slow down OA disease progression.^[^ ^2^, ^3^ ^]^ In contrast, hyper‐physiological loading, as seen with post‐traumatic injuries such as articular fracture, meniscal tear, or rupture of the anterior cruciate ligament, concomitant with altered joint kinematics, is a major risk factor for the onset and progression of OA.^[^ ^4^ ^]^ Both physiological and hyper‐physiological loading results in alterations in the structural composition of the cartilage extracellular matrix.^[^ ^1^ ^]^ These findings suggest that the balance of chondrocyte anabolic and catabolic processes that maintain cartilage homeostasis is tightly regulated by biomechanical cues.

Chondrocytes reside in a pericellular matrix (PCM), which modulates the transduction of mechanical cues from the extracellular matrix (ECM) towards the chondrocyte. The PCM for that matter contains specific molecular components such as collagen 6 (COLVI) that are known to regulate the biomechanical environment of the chondrocyte, e.g., via calcium signaling in response to mechanical stress.^[^ ^5^, ^6^, ^7^ ^]^ This is further evidenced by knock‐out of a COLVI sub‐unit alpha 1 in a murine model, which causes an osteoarthritic phenotype through dysregulation of mechanotransduction.^[^ ^5^, ^8^ ^]^ Nonetheless, the lasting effects of dysregulated mechano‐transduction after injurious mechanical loading conditions on the cellular phenotype of the chondrocyte is, however, less well studied and remains unclear.

Here we report the identification of a *COL6A3* missense variant through exome sequencing of patients with generalized OA (Genetics osteoArthritis Research and Progression (GARP) study).^[^ ^9^ ^]^ *COL6A3*, coding for one of the monomeric sub‐units of COLVI^[^ ^10^ ^]^ that resides in and interacts with other PCM proteins,^[^ ^11^, ^12^ ^]^ is likely involved in the regulation of cartilage structural composition and mechanical properties. We hypothesized that the identified *COL6A3* variant would perturb mechano‐transduction in response to hyperphysiologic loading, thereby affecting the chondrocyte cellular phenotype. Recently, OA disease modeling platforms utilizing human induced pluripotent stem cells (hiPSCs) have been used to facilitate the interrogation of these pathogenic variants.^[^ ^13^ ^]^ To gain an understanding of the role of COLVI during chondrogenesis and chondrocyte function in exposure to environmental stressors, we studied this variant in an in vitro model of OA using hiPSC‐derived cartilage. Hereto, we employed genetically *COL6A3*‐edited hiPSCs to an established in vitro cartilage organoid model, and these neocartilage organoids were exposed to hyper‐physiological mechanical loading conditions. To study the effects of this variant on the chondrocyte phenotype in response to hyper‐physiologic mechanical loading conditions, we characterized downstream molecular pathways based on RNA transcriptome wide profiles (mRNA and lncRNA) to identify changes in the chondrocyte phenotypic state and associated regulatory epigenetic mechanisms, for druggable target discovery.

## 2 Results

### 2.1 Identification of a Damaging Variant in *COL6A3*

Whole exome‐sequencing was applied to a Caucasian OA patient of Dutch ancestry at the age of 61 affected predominantly with symptomatic OA at multiple sites (Genetics osteoArthritis Research and Progression (GARP) study).^[^ ^9^ ^]^ The exome sequencing resulted in the detection of 81416 genetic variants, after which a prioritization scheme was followed to identify dominant pathogenic variants as previously described.^[^ ^13^, ^14^ ^]^ In short, first, we selected novel variants in in‐house whole genome sequencing projects (*N* = 222) and the BBMRI‐Genome of the Netherlands project (GoNL, *N* = 473).^[^ ^14^ ^]^ And second, damaging missense variants were selected based on the prediction of sorting intolerant from tolerant (SIFT).^[^ ^15^, ^16^ ^]^ This prioritization generated a dataset of 38 novel coding variants that were predicted to have a functional impact on the protein of each identified coding variant (Table S1, Supporting Information). And third, these 38 novel coding variants were then further prioritized based on their expression patterns in disease‐relevant tissue by means of in silico analysis of a previously published RNA‐sequencing dataset containing 58 paired preserved and lesioned OA cartilage and subchondral bone samples of patients undergoing joint replacement surgery.^[^ ^17^, ^18^ ^]^ We have prioritized genes that were both highly expressed in joint tissues since these genes likely determine characteristics of the tissue and/or the cellular phenotype of chondrocytes and were differentially expressed between preserved and lesioned OA tissues (cartilage) since these genes mark the OA pathophysiological phenotype of chondrocytes. This resulted in the prioritization of two heterozygous variants affecting protein function of genes that are highly expressed in cartilage and differentially expressed between lesioned and preserved cartilage and bone; *MTHFR* (c.1667C>T, p.Pro597Leu, P597L); cartilage: FC = 0.84, FDR = 0.04, bone: not detected) and *COL6A3* (c.4510C>T, p.Arg1504Trp, R1504W); cartilage: FC = 1.75, FDR = 2.25 × 10^−5^, bone: FC = 1.69, FDR = 0.042).^[^ ^18^ ^]^ *MTHFR* encodes for methylenetetrahydrofolate reductase, which is involved in folate metabolism.^[^ ^19^ ^]^ *COL6A3* encodes for collagen VI subunit A3, which together with collagen VI subunit A1 and A2 forms a triple helical COLVI, a primary component of the cartilage PCM, and interacts with other proteins such as fibronectin and hyaluronan.^[^ ^11^, ^12^, ^20^ ^]^ We then prioritized the *COL6A3* variant because of its relevance to OA and involvement in mechano‐transduction.^[^ ^5^ ^]^ The identified *COL6A3* variant is located in the 3^rd^ N‐terminal VWA domains that are known to protrude away from the triple helical structure. Moreover, upon exploring the effect of the R1504W variant on protein function by additional in silico tools using sequence homology and evolutionary conservation; Polyphen 2,^[^ ^21^ ^]^ Provean, and PANTHER‐PSEP^[^ ^22^ ^]^ it was shown that predicted damaging effect on protein function was with high confidence (Table S2, Supporting Information). Furthermore, the in silico tools i‐mutant V2.0 and MUpro, using support vector machines, predicted that the R1504W variant decreases the stability of the protein (Table S2, Supporting Information). To gain an understanding of the effects of aberrant COLVI function on chondrocyte phenotype in interaction with mechanical loading, we selected this predicted damaging variant for further study.

### 2.2 Introducing the Variant in hiPSCs

To investigate the effects of the aberrant COLVI on matrix deposition and molecular pathways, a gene‐edited human induced pluripotent stem cell (hiPSC)‐derived neocartilage organoid model was generated. We employed CRISPR‐Cas9 single‐stranded oligonucleotide‐mediated homology‐directed repair in hiPSCs to attain. heterozygous modification of rs144223596 (c.4510C>T) in an isogenic background, which was confirmed by Sanger sequencing (Figure S2, Supporting Information). To further prevent bias in our results, we have screened for potential off‐target effects using CRISPOR resulting in four sites that could be affected, be it with low likely‐hood^[^ ^23^ ^]^ (Figure S2F, Supporting Information). We then confirmed by Sanger sequencing that these sites were not affected by off‐target CRISPR‐Cas9 single‐stranded oligonucleotide‐mediated homology‐directed repair (Figure S2G, Supporting Information). Next, the *COL6A3*‐edited and the unedited isogenic control hiPSCs were differentiated into chondrocytes using a previously established chondrogenic differentiation protocol.^[^ ^24^ ^]^ In short, hiPSCs followed a step‐wise differentiation protocol via mesodermal lineage differentiation towards chondroprogenitor cells.^[^ ^24^ ^]^ These cells were then dissociated, and chondrogenesis was initiated in our organoid pellet model.

### 2.3 Characterization of the Matrix of Isogenic Control and *COL6A3* Variant hiPSC‐Derived Cartilage Organoids

**Figure 1:**
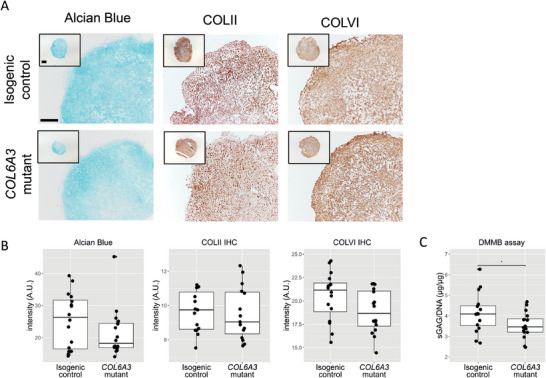
Effect of the variant on neocartilage matrix deposition. A) Representative images of Alcian blue staining marking sulfated glycosaminoglycans (sGAGs) and immunohistological staining of collagen II (COL II) and collagen VI (COLVI). Scale bar 200 µm. B) Quantification of Alcian blue, COLII, and COLVI in isogenic control and *COL6A3*‐variant neocartilage organoids showed no significant effect of the variant (*n* = 16). C) Quantification of sGAG deposition in neocartilage organoids. sGAG deposition in COLVI‐variant neocartilage organoids is reduced in comparison to isogenic controls (beta = 0.55 ± 0.28, *t* = 1.96, *P* = 4.90 × 10^−2^, *n* = 16). Statistics: B,C) *P* values were attained using a generalized linear model with B) intensity (for immunostaining) and C) (sGAGs/DNA) as dependent variable and genotype as independent variable. The box plots represent 25th, 50th, and 75th percentiles, and whiskers extend to 1.5 times the interquartile range. Individual samples are depicted by black dots in each graph. *P < 0.05.

Successful differentiation towards chondrocytes and the production of neocartilage was confirmed by protein staining of collagen II (COLII), collagen VI (COLVI), and sulfated glycosaminoglycans (sGAGs) (**Figure** **1**A), and comparable to isotype controls as shown previously.^[^ ^25^ ^]^ COLII and COLVI deposition was not affected by the variant as measured by staining intensity (Figure 1B). However, quantification of sGAG deposition, using the dimethyl methylene blue (DMMB) assay normalized to DNA content, showed a significant reduction in the COLVI‐variant compared to the isogenic control neocartilage organoids (Figure 1C). Next, the isogenic and COLVI‐variant neocartilage organoids were characterized by targeted gene expression analysis using RT‐qPCR of markers relevant for cartilage homeostasis which were previously shown to be mechano‐responsive (Table S3, Supporting Information).^[^ ^25^ ^]^ The *COL6A3* variant reduced expression of the catabolic marker *ADAMTS5*, while also reducing expression of the anabolic markers *COL2A1* as well as *ACAN*.

### 2.4 Transmission Electron Microscopy of the Cartilage Neomatrix

**Figure 2:**
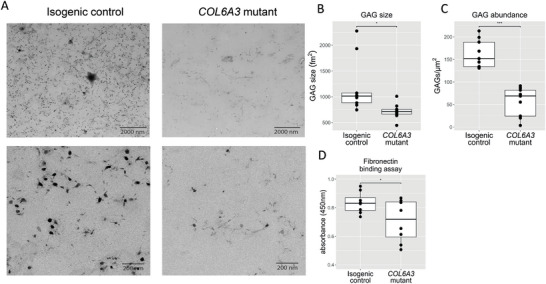
The collagen VICOLVI variant reduces sGAG aggregate size and reduces binding to fibronectin. A) TEM image of isogenic control (left) and a *COL6A3*‐variant (right) neocartilage organoid. B) Using a deep‐learning algorithm, the sGAG aggregate size was measured, showed a reduction in the *COL6A3*‐variant neocartilage organoids (beta = −479.7 ± 184.4, *t* = −2.60, *P* = 1.93 × 10^−2^). C) GAG amount was decreased in the *COL6A3*‐variant neocartilage organoids (beta = −106.14 ± 14.95, *t* = −7.10, *P* = 2.52 × 10^−6^). D) Solid‐phase binding assay with full‐length fibronectin‐coated wells and wild‐type and R1504W variant COLVI. R1504W showed a reduced binding to fibronectin (beta = −0.13 ± 0.06, *t* = −2.17, *P* = 4.37 × 10^−2^, *N* = 8). This assay did not show binding of COLVI to hyaluronan. Statistics: B–D) *P* values were attained using a generalized linear model, with sGAG size, sGAGs mm^−2^, or absorbance at 450 nm as a dependent variable and genotype as an independent variable. The box plots represent 25th, 50th, and 75th percentiles, and whiskers extend to 1.5 times the interquartile range. Individual samples are depicted by black dots in each graph. *P < 0.05, ***P < 0.001.

To further investigate the effect of the variant on the structural properties of the PCM and ECM, we performed transmission electron microscopy. As shown in **Figure** **2**A, there was a reduced abundance of sGAG‐like structures in the *COL6A3*‐variant neocartilage organoids versus the isogenic control. Consistent with the DMMB assay, upon performing quantitative analysis of these sGAG‐like structures using a deep learning algorithm,^[^ ^26^ ^]^ we confirmed that variant COLVI cartilage had significantly decreased aggregate size (Figure 2B) and sGAG abundance (Figure 2C).

### 2.5 Binding of Isogenic Control and *COL6A3* Variant COLVI to Pericellular Matrix Proteins

The identified R1504W variant is located in the N‐terminal domain of *COL6A3* coding for a von Willebrand Factor A domain, which is involved in binding two important constituents in cartilage PCM and ECM, fibronectin and hyaluronan.^[^ ^12^, ^13^, ^20^ ^]^ Arginine at this position in the *COL6A3* protein is evolutionary highly conserved, indicating that an amino acid change at this position is likely to affect protein function (Figure S1, Supporting Information). Thus, we hypothesized that the change from a polar arginine to a non‐polar tryptophan affects the interaction between COLVI and PCM/ECM proteins. To this end, we performed a fibronectin and hyaluronan solid‐phase binding assay with wild‐type and variant COLVI, which was extracted from the chondrogenic differentiation medium of neocartilage organoids. Results, as shown in Figure 2D, demonstrated that binding to fibronectin was reduced for variant COLVI. It must be noted that this binding could be indirect, via other proteins in the medium. However, we could not detect any binding of wild‐type nor variant COLVI to hyaluronan in our assay.

### 2.6 Effect of Missense *COL6A3* Variant on the Transcriptomic Landscape

**Figure 3:**
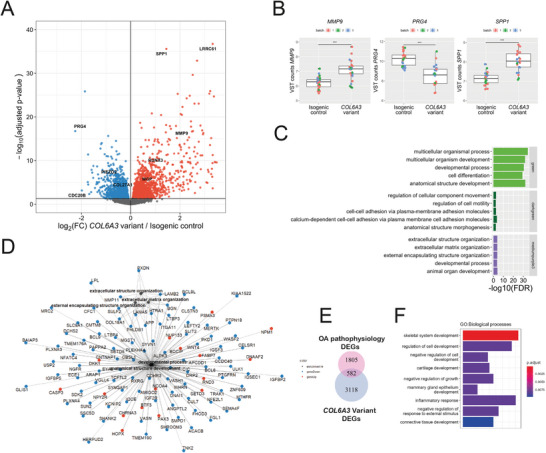
Transcriptomic profile in presence of the *COL6A3* variant. A) Volcano plot showing the differentially expressed genes (DEGs) in response to the *COL6A3* variant (*n* = 25–26). Red dots denote DEGs with an FDR < 0.05 that are upregulated, and blue dots represent DEGs that are downregulated, as determined by DESeq2 analysis. B) Notable examples of DEGs between isogenic control and *COL6A3*‐variant neocartilage pellets. The box plots represent 25th, 50th, and 75th percentiles, and whiskers extend to 1.5 times the interquartile range. Individual samples are depicted by black dots in each graph. ***FDR < 0.001. C) Overrepresentation enrichment analysis (KEGG, REACTOME, GO biological processes) of the top 3 weighted gene coexpression network analysis (WGCNA) coexpression modules where the first principal component is significantly associated with the *COL6A3* variant. D) Pathway—gene network of the enrichment analysis of the mediumpurple3 cluster. Lines depict the relationship between the genes and the pathways determined by enrichment analysis. Blue dots depict downregulated DEGs with the *COL6A3* variant, while red dots depict upregulated DEGs with the *COL6A3* variant. E) Overlap between the *COL6A3* variant DEGs and differential expression between lesioned and preserved cartilage from a previously published dataset (RAAK study, Ramos et al.,^[^ ^17^ ^]^). F) Over‐representation enrichment analysis of overlapping DEGs between the variant and lesioned versus preserved cartilage. Count depicts the number of genes that are categorized to each pathway. Statistics: A,B) negative binomial generalized linear model, C,D,F) Fisher exact test.

Next, being one of the most sensitive and informative measures of cellular responses to genetic perturbations, we performed mRNA sequencing, to determine the downstream effects of these changes in the PCM and ECM secondary to the damaging *COL6A3* variant. Multifactorial analysis, using surrogate variable analysis (SVA) correction (Figure S3, Supporting Information),^[^ ^27^ ^]^ revealed 3700 significant DEGs between the *COL6A3* variant and isogenic controls (FDR < 0.05) (**Figure** **3**A; Table S4, Supporting Information). Among these genes, 55% were upregulated and 45% were downregulated. Notable highly significant DEGs were related to development and cartilage metabolism, with the downregulation of structural PCM and ECM proteins, such as *COL27A1* and *PRG4*, increased catabolic activity, such as *MMP9*, and ECM mineralization, such as *SPP1* (Figure 3A,B).

To determine the biological processes associated with the *COL6A3* variant, we performed a weighted gene co‐expression network analysis (WGCNA) on the RNA‐sequencing data set. This resulted in the detection of 20 distinct co‐expression networks (Figure S4, Supporting Information). Multifactorial regression analysis revealed 10 co‐expression networks with the first principal component significantly associated with the *COL6A3* genotype (Figure S4, Supporting Information). The top 3 most significant co‐expression networks associated with the *COL6A3*‐variant genotype were enriched for developmental processes, cell–cell adhesion/anatomical structure morphogenesis, and ECM organization (Figure 3C; Table S5, Supporting Information). Enrichment of ECM organization again underlines the detrimental effects of the *COL6A3* variant on the development of cartilage, affecting genes such as *LAMA5*, *LAMB2*, and *COL18A1* that code for structural PCM components (Figure 3D). While enrichment of cell‐cell adhesion pathways suggests that the variant also affects mechano‐transduction via altered cell–cell adhesion by downregulation of cadherin‐associated genes, such as *CDH18*, *CDH6*, *PTGER4*, *MAPK14*, and *ADAMTS5* (Figure S5, Supporting Information). Of note is that *ADAMTS5* is downregulated in the *COL6A3* variant while quantification of sGAGs, the target peptides of ADAMTS5 cleavage, are reduced at both the gene level (*ACAN*, Table S3, Supporting Information) and protein level (Figures 1C and 2A–C).

To study the relevance of the *COL6A3* variant to OA pathophysiology, we determined the overlap of differentially expressed genes with the *COL6A3* variant and those previously reported between lesioned and preserved cartilage from OA patients who underwent a joint replacement surgery. The latter marks the heterogeneous overall pathophysiologic state of OA chondrocytes. This revealed a subset of 582 overlapping DEGs (Table S6, Supporting Information). We then performed pathway analysis on this subset of genes (Figure 3F). The most significant enriched pathway was skeletal system development, including transcription factors (*RUNX3*, *TNFRSF11B*), ECM components (such as *COL27A1*, *COL11A2*), and anabolic factors (such as *IGF1*, *BMP3*). Well‐known pathways related to inflammation “inflammatory response” and “acute inflammatory response” were also enriched containing inflammatory cytokines (*IL31A*) and general inflammatory response factors (*PTGES*, *HLA‐e*). Also, the “ossification” pathway was enriched, containing OA risk genes (*SPP1*, *TNC*, and *MGP*). Genes related to cartilage metabolism and mechano‐sensing were confirmed by quantitative reverse transcription polymerase chain reaction (RT‐qPCR) (Table S7, Supporting Information). Together, this data suggests that the *COL6A3* variant results in downstream expression changes, in part describing the osteoarthritis pathophysiology.

The stranded RNA sequencing allowed us to confirm the genotyping as performed with Sanger sequencing (Figure S1, Supporting Information). This showed a dispersion of the variant allele frequencies (VAFs) in the included CRISPR‐Cas9 edited samples with an average proportion of 0.70 towards the damaging *COL6A3* variant allele (Table S8, Supporting Information). To explore possible dose‐response effects of the dispersed *COL6A3* variant frequency, the VAF was considered as a covariate and analyzed for differential expression. This revealed a robust dose‐response effect (FDR < 0.05) of the damaging *COL6A3* variant on downstream gene expression in 2728 genes out of the 3700 DEGs (74%), including genes such as *MMP9*, *PRG4*, and *SPP1* (Table S9 and Figure S7, Supporting Information).

### 2.7 Effect of Mechanical Loading on the Transcriptomic Landscape

**Figure 4:**
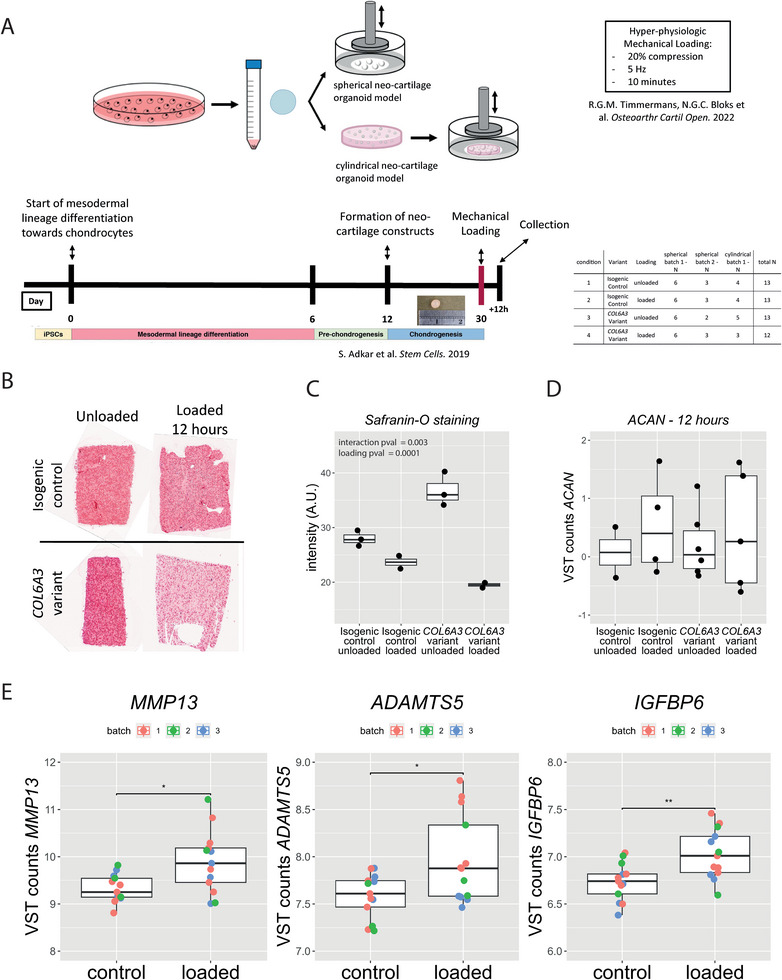
Chondrogenic models to study the effects of aberrant COLVI function in interaction with hyper‐physiologic mechanical loading conditions. A) hiPSCs in which the R1504W variant was introduced using CRISPR‐Cas9 genome editing. These cells were differentiated using an established differentiation protocol to produce neocartilage organoids. Two different organoid models were employed and jointly analyzed; 1‐A) Spherical pellet model harnessing the original matrix produced by the hiPSCs. 2‐A) Cylindrical organoid model in which the hiPSC‐derived chondrocytes were embedded in an agarose construct, ideally suited for testing the effects of mechanical loading conditions. These constructs were both exposed to hyper‐physiological loading conditions, after which the organoids were harvested for downstream analysis. B) Safranin “O” staining at 12 h after hyperphysiological mechanical loading. C) Safranin “O” staining intensity at 12 h after hyper physiological mechanical loading (*n* = 2–3). D) RT‐qPCR data of ACAN and *COL2A1* expression at 12 h and 4 d after hyperphysiological mechanical loading (*n* = 2–6). E) Upregulation of post‐traumatic markers in isogenic control samples measured by RNA‐sequencing (*n* = 13). Statistics: C,D) Generalized linear model. E) Negative binomial generalized linear model. The box plots represent 25^th^, 50^th^, and 75^th^ percentiles, and whiskers extend to 1.5 times the interquartile range. *FDR < 0.05.

**Figure 5:**
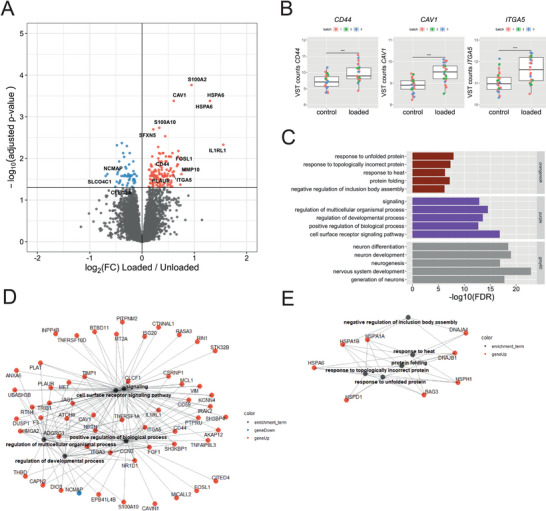
Transcriptomic profile in response to hyper‐physiologic mechanical loading conditions. A) Volcano plot of differentially expressed genes (DEGs) in response to hyperphysiologic loading conditions (*n* = 25–26). Red dots denote differentially DEGs with an FDR < 0.05 that are upregulated, and blue dots represent DEGs that are downregulated as determined by DESeq2 analysis. B) Notable examples of mechano‐sensor genes upregulated in response to hyper‐physiological mechanical loading conditions. The box plots represent the 25^th^, 50^th^, and 75^th^ percentiles, and the whiskers extend to 1.5 times the interquartile range. Individual samples are depicted by black dots in each graph. ***FDR < 0.001. C) Overrepresentation enrichment analysis (KEGG, REACTOME, GO biological processes) of the top 3 weighted gene co‐expression network analysis (WGCNA) co‐expression modules of which the first principal component is significantly associated with hyper‐physiologic mechanical loading conditions. D,E) Gene‐pathway network of the enrichment analysis of D) the purple and the orangered4 € coexpression network where lines depict the relationship between the genes and the pathways determined by enrichment analysis. Blue dots depict downregulated DEGs in response to hyperphysiologic mechanical loading conditions, red dots depict upregulated DEGs in response to hyper‐physiologic mechanical loading conditions. Statistics: A,B—negative binomial generalized linear model, C–E—Fisher exact test.

Next, we characterized the effect of hyperphysiologic loading conditions as previously defined^[^ ^28^ ^]^ on these neocartilage organoids (**Figure** **4**A). Hereto, two different organoid models were applied and jointly analyzed; cylindrical constructs in which hiPSC‐derived pellets were digested to obtain single‐cell chondrocytes that were then encapsulated in an agarose gel, and spherical constructs with neocartilage deposited by hiPSC‐derived chondrocytes. Both these organoid models were exposed to hyper‐physiologic mechanical loading conditions (20% sinusoidal peak‐to‐peak strain at 5 hz for 10 min) cartilage organoids were harvested at 12 hours post loading. As shown in Figure 4B and as measured by of Safranin‐O staining, hyper‐physiological loading resulted in a moderately reduction of proteoglycan content whereas in the *COL6A3* mutated organoids large reduction was observed. These observations were however not confirmed by qPCR data (Figure 4B; Table S7, Supporting Information). Induction of a post‐traumatic response by hyper‐physiologic loading conditions was confirmed by upregulation of *MMP13, ADAMTS5*, and *IGBP6* gene‐expression (Figure 4E). This was in line with previous results in an ex vivo model measuring genome‐wide expression after injurious mechanical loading.^[^ ^25^, ^29^, ^30^ ^]^ To obtain in depth insight into phenotypic changes of the chondrocyte in response to hyper‐physiological loading conditions, transcriptome wide activity was subsequently measured by RNA sequencing. Joint multifactorial analyses of the two models revealed 177 DEGs (FDR < 0.05) between unloaded and mechanically loaded organoids, of which 74% were upregulated and 26% were downregulated (**Figure** **5**A and Table S10, Supporting Information). Notable genes were, amongst others; *CD44*, *CAV1*, *ITGA5* (Figure 5B), which are all genes encoding for mechano‐sensors involved in OA pathophysiology. To determine the biological processes affected by hyper‐physiologic mechanical loading WGCNA co‐expression networks were associated with mechanical loading, revealing 5 significantly associated co‐expression networks (Figure S4 and Table S6, Supporting Information). The top 3 most significantly associated modules were enriched for developmental and signaling processes, stress responses, and neuronal pathways (Figure 5C). While enrichment of signaling responses containing genes such as *IL1RL1* and *FOSL1* show an adaptive response to hyper‐physiologic mechanical loading conditions (Figure 5D), enrichment of stress responses with genes such as *HSPA1B* and *DNAJA4* underlines the damaging effects of the hyper‐physiologic mechanical loading conditions (Figure 5E).

### 2.8 Effect of the *COL6A3* Missense Variant on the Response to Hyperphysiological Mechanical Loading Conditions

**Figure 6:**
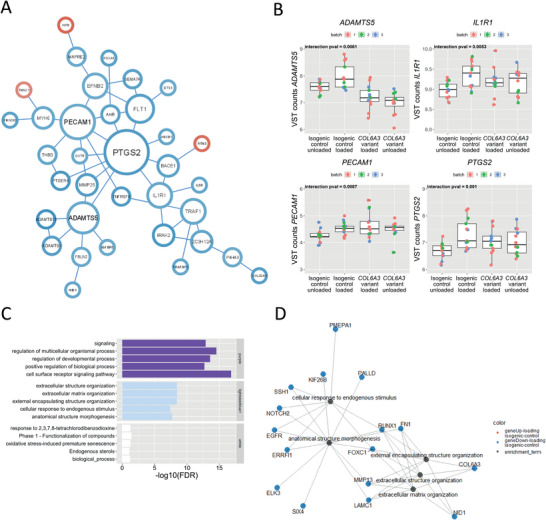
The *COL6A3* variant affects the biological response to hyper‐physiologic loading conditions. A) Protein–protein interaction (PPI) network based on the STRING‐DB of genes that show an interaction (*P <* 0.01) between the *COL6A3* variant and hyperphysiological mechanical loading conditions, as determined by DESeq2 analysis. Red circles denote an increased response to hyperphysiological mechanical loading due to the *COL6A3* variant, while blue circles denote a reduced response to mechanical stress due to the *COL6A3* variant (only showing connected nodes). Relative node size depicts number of connections for each gene within the network (*n* = 25–26). B) Examples of central DEGs in the PPI that are related to an inflammatory response. The box plots represent 25th, 50th, and 75th percentiles, and whiskers extend to 1.5 times the interquartile range. Individual samples are depicted by black dots in each graph. C) Gene enrichment analysis of WGCNA hubs significantly related to the interaction between the *COL6A3* variant and the response to hyperphysiological mechanical loading conditions. D) Pathway—gene network of the enrichment analysis of the purple cluster. Lines depict the relationship between the genes and the pathways determined by enrichment analysis. Blue and red dots depict, respectively, downregulated and upregulated DEGs in response to hyperphysiological loading conditions in the isogenic control neocartilage organoids. Statistics: A) log‐likelihood score for PPI, A,B) negative binomial generalized linear model for differential expression analysis, C, D)Fisher exact test.

Next, the effects of the damaging *COL6A3* variant on the response to hyperphysiological mechanical loading were investigated. To this end, a multifactorial analysis was performed resulting in a set of 135 genes with a significant interaction effect (*P* < 0.01) indicating that the variant affected the response to hyperphysiological mechanical loading (Table S11, Supporting Information). Of these 135 genes, 70 proteins show a significant protein‐protein interaction (PPI) (FDR < 0.05) as determined by STRING‐DB (**Figure** **6**A). Notable is that highly connected genes in this PPI network such as; *PTGS2*, *IL1R1, IRAK2, PECAM1*, and *ADAMTS5* are all related to catabolic and inflammatory signaling. *PTGS2* is known to be regulated by TRPV4 signaling in response to mechanical stress and is involved in inducing an inflammatory response to stimuli.^[^ ^31^ ^]^ *IL1R1* codes for the receptor of interleukin‐1, one of the key inflammatory markers in OA, and thus is involved in inflammatory signaling, while *IRAK2* encodes for the interleukin‐1 receptor‐associated kinase 2, which is involved in interleukin‐1 (IL‐1) induced upregulation of NF‐κβ.^[^ ^32^ ^]^ Upon performing stratified analysis, it was shown that the interaction effect was particularly caused by an upregulation of gene expression observed in isogenic controls in response to hyperphysiological loading conditions, that appeared absent in the *COL6A3* variant neocartilage organoids (Figure 6B; Table S12, Supporting Information).

To study the biological processes in which the response to hyper‐physiological loading differed between the isogenic control and the *COL6A3* variants, associate this interaction effect with the principal component of each detected co‐expression network. This revealed three distinct co‐expression networks associated with the interaction effect, which were enriched for processes related to signaling, ECM organization, and general biological processes (Figure 6C). Enrichment of pathways related to signaling shows an aberrant response in genes such as *INHBA* which acts downstream of TRPV4 calcium channels, *CAV1*, *SEMA7A*, and *MMP3*, which are all genes that respond to mechanical loading in isogenic controls, with an absent loading effect in the *COL6A3* variant (Table S5, Supporting Information). Again, the enrichment of processes related to ECM formation and organization, containing genes such as; *MMP13, RUNX1, FN1, LAMC1, EGFR*, and *PMEPA1* (Figure 6D), underlines the effect of an impaired repair response to hyper‐physiological mechanical loading conditions in the *COL6A3* variant. Also, the enrichment of general biological processes including *IL1R1* highlights the aberrant response to mechanical loading in the *COL6A3* variant neocartilage organoids.

Finally, we studied longer term effect of the response to hyper‐physiological mechanical loading conditions in the *COL6A3* variant compared to the isogenic controls. To this end, gene expression analysis and safranin‐o staining were performed 12 hours and 4 days after mechanical loading in both models (Figure 4). As shown in Figure S8A (Supporting Information), the transient inflammatory response appeared in remission in isogenic controls and was still absent in the *COL6A3* mutated chondrocytes at day 4 after hyper‐physiological mechanical loading. Furthermore, as shown in Figure S8B,C (Supporting Information), a consistent pattern in the proteoglycan content, as measured by safranin‐o staining is shown in both models. More specifically, in the isogenic controls, the proteoglycan content 12 h after loading dropped and was restored at days 4 after loading to levels observed in the unloaded isogenic controls. In the *COL6A3* variant, however, a strong reduction of proteoglycan content was observed at 12 h after hyper physiological loading that was not fully restored at day 4. These observations were confirmed by *ACAN* gene expression in the cylindrical model, in contrast to the spherical organoid model (Figure S8D, Supporting Information). Regarding the ongoing anabolic responses, as measured by *COL2A1* expression, it was shown in the isogenic control that the initial reparative anabolic response 12 h after hyper physiological loading has turned to unloaded “steady state” levels 4 d after hyper physiological mechanical loading. In the *COL6A3* mutated chondrocytes, however, it was shown in both models that 4 days after loading *COL2A1* expression is still increased, highlighting an activated chondrocyte state (Figure S8D, Supporting Information). Together, this data suggests that after hyper physiological mechanical cues the *COL6A3* neocartilage organoids are subject to long‐term differential effects as compared to the isogenic controls.

### 2.9 Epigenetic Regulation by *MIR31HG* as a Driver of the Interacting Effect between the *COL6A3* Missense Variant and the Hyperphysiological Mechanical Loading Induced Stress Response

**Figure 7:**
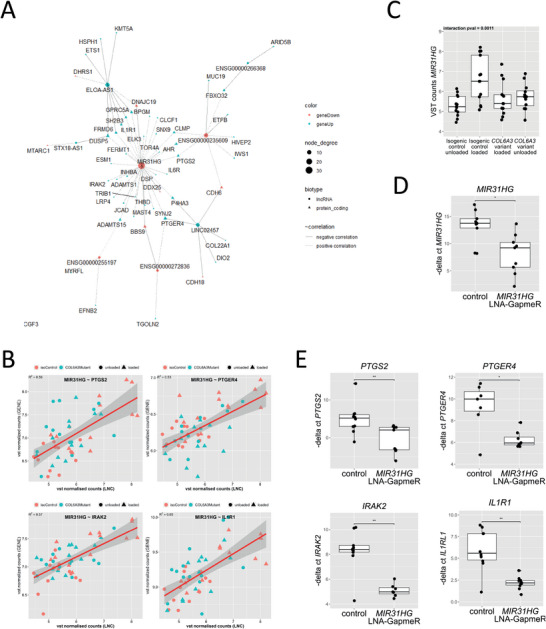
Druggable target discovery of *MIR31HG* as a regulator of the inflammatory response to hyperphysiological mechanical loading. A) *MIR31HG* as a central hub in a lncRNA–protein coding correlation network (FDR < 0.05, *r* ^2^ > 0.5) of genes showing a significant interaction between hyper‐physiological mechanical loading and the *COL6A3* variant. B) Individual gene expression plot of VST normalized gene expression plot of *MIR31HG* showing interaction effect between mechanical loading and the *COL6A3* variant (*n* = 26). C) Correlation plots of VST normalized counts of protein coding genes and *MIR31HG* (*n* = 13 per condition). D) Consistent downregulation of *MIR31HG* by LNA‐GapmeR (FC = 0.02, *P* = 6.4 × 10^−3^, *n* = 10). E) *MIR31HG* inhibition using a LNA‐GapmeR consistently downregulates *PTGS2* (FC = 0.03, *P* = 9.14 × 10^−3^), *IRAK2* (FC = 0.11, *P* = 4.44 × 10^−4^), *PTGER4* (FC = 0.12, *P* = 9.32 × 10^−3^), *IL1R1* (FC = 0.07, *P* = 5.71 × 10^−3^) (*n* = 10). Statistics: A,B) Spearman's rank correlation, C) negative binomial generalized linear model, D,E) generalized linear model. The box plots represent the 25th, 50th, and 75th percentiles, and the whiskers extend to 1.5 times the interquartile range. Individual samples are depicted by black dots in each graph. *P < 0.05, **P < 0.01, ***P < 0.001.

Finally, we aimed to find key regulators behind the interacting effect of the *COL6A3* variant and the hyper‐physiological mechanical loading‐induced stress response. We chose to integrate long‐non‐coding (lnc)RNAs as epigenetic regulatory elements, as these previously have been shown to regulate gene expression in OA pathology.^[^ ^33^ ^]^ To this end, expression analysis of lncRNAs as annotated by the GENCODE V41^[^ ^34^ ^]^ was performed. Multifactorial analysis resulted in a significant interaction effect between the *COL6A3* variant and the response to hyper‐physiological mechanical loading in a set of 17 lncRNAs (*P* < 0.01) (Table S13, Supporting Information). Notable examples are *LINC00574* as well as *MIR31HG*, which were previously reported as being higher expressed in lesioned cartilage compared to preserved cartilage from OA patients who underwent joint replacement surgery.^[^ ^33^ ^]^ To screen for potential regulatory effects of lncRNAs on protein‐coding gene expression, we integrated the lncRNA data with the protein‐coding RNA expression data. This resulted in a correlation network of 12 unique lncRNAs with 55 unique protein‐coding genes for a total of 72 significant correlations (*r* ^2^ > 0.5, FDR < 0.05) (**Figure** **7**A). Of particular interest was *MIR31HG* as it appears as a central interacting node in the correlation network, accounting for 32 out of the 72 significant correlations, interacting with stress response and inflammation related genes such as *PTGS2, PTGER4, IRAK2*, and *IL1R1*(Figure 7B). Genomically, *MIR31HG* is located near *IFNA8*, *IFNA1* and *IFNE*. and potentially directly interacts with its inflammatory signaling. Additionally, *MIR31HG* strongly responds to mechanical loading in isogenic controls, whereas this response is absent in *COL6A3* variants. Hence, given *MIR31HG* is a central interaction node in the correlation network (Figure 7A,B) and its lack of response to mechanical loading in *COL6A3* variants (Figure 7C) we hypothesized that it regulates these inflammatory genes. Thus, finally, we validated this regulatory role of *MIR31HG* in the expression of these genes by transfection of *MIR31HG* targeting LNA‐GapmeR in primary chondrocytes. As shown in Figure 7d this resulted in a significant knock‐down of *MIR31HG* expression in comparison to a nontargeting LNA GapmeR. Subsequently, we have measured the expression of stress response and inflammation‐related genes in response to *MIR31HG* targeting LNA‐GapmeRs, which showed significant downregulation of *PTGS2, PTGER4, IRAK2*, and *IL1R1* (Figure 7E). Together these results provide experimental validation that *MIR31HG* is a key regulator of the initial stress response to hyper‐physiologic mechanical loading and that this regulatory action of *MIR31HG* in the damaging *COL6A3* variant neocartilage organoids is absent.

## 3 Discussion

In the current study, we identified a missense variant in *COL6A3* (c.4510C>T, R1504W) in a subject of the GARP study affected with symptomatic OA in two or more joint sites.^[^ ^9^ ^]^ By introducing this damaging variant in hiPSCs using CRISPR‐Cas9 genome engineering and employing these cells in two different established 3D in vitro neocartilage organoid models, we showed that the variant decreases cartilage matrix integrity, as reflected by a reduction in abundance and size of sGAGs. Moreover, by subsequently isolating mutated COLVI protein, we showed that it had reduced binding to FN. Being both important and particular proteins of the chondrocyte PCM, the reduced binding is likely affecting PCM function. Analysis of the transcriptome‐wide gene expression changes with the *COL6A3* variant, showed overlap to those observed with OA‐pathophysiology. Together these data indicated that the *COL6A3* variant is likely affecting the propensity of chondrocytes to enter an osteoarthritic state, secondary to its effect on PCM function. By subsequently exposing the neocartilage organoids to hyper‐physiological mechanical stress, we demonstrated that *COL6A3* variant in chondrocytes abolished the characteristic upregulation of inflammatory signaling after mechanical loading^[^ ^25^, ^35^, ^36^ ^]^ with *PTGS2*, *PECAM1*, and *ADAMTS5*, as most central genes. We also showed that an aberrant chondrocyte phenotype in the *COL6A3* variant persisted at least 4 days. Finally, by integrating epigenetic regulation of protein coding gene expression we identified lncRNA *MIR31HG* as a key regulator of the characteristic inflammatory signaling observed in response to mechanical loading, a response that was abolished in the *COL6A3* variant. Taken together, our findings suggest that the identified variant in *COL6A3* resulted in impaired binding between COLVI and the PCM protein FN. Secondary to these alterations in PCM function the initial stress response to hyper‐physiologic mechanical loading conditions was abolished that in turn affected the propensity of the chondrocyte to enter an OA disease state.

By integration of lncRNA's driven epigenetic regulation of protein‐coding genes, we were able to identify *MIR31HG* as a key regulator in the aberrant response to mechanotransduction in the *COL6A3* variants. *MIR31HG* expression has previously been reported in OA pathophysiology.^[^ ^33^ ^]^ Mechanistically, in other cell types, *MIR31HG* has been shown to activate the expression of similar inflammatory genes such as *IL1R1*, *PTGS2*, and *TNFRSF11B*,^[^ ^37^ ^]^ but also genes that have previously been linked to damaging loading in cartilage such as *IGFBP5* and *IGFBP7*, via promoting phosphorylation of *YBX*1, thereby initiating translation of *IL1A*. This further underlines the relevance of *MIR31HG* for osteoarthritis. We would like to postulate that the loss of the initial response of *MIR31HG* to mechanical loading, and hence, the loss of the transient inflammatory response as marked by, amongst others, *PTGS2* and *IL1R1*, inhibit subsequent repair of cartilage. By studying a damaging *COL6A3* variant in two hiPSC‐derived neocartilage organoid models, we have been able to study in detail the downstream chondrocyte mechano‐biologic effects to hyper‐physiological mechanical stress. Nonetheless it should be noted that upon checking the heterozygous introduction of the damaging *COL6A3* variant by genotyping the RNA sequencing data, an allelic imbalance of the *COL6A3* variant relative to the reference allele was observed. This predominance of *COL6A3* variant expression could indicate increased stability of the *COL6A3* variant mRNA allele or alternatively could be caused due to an unrecognized mixed population of homo‐ and heterozygous hiPSC clones. Irrespectively, the allelic imbalanced expression of the *COL6A3* variant confirmed a dose‐response effect of the presence *COL6A3* variant in 74% of identified DEGs including notable genes such as *SPP1*, *MMP9*, and *PGR4*. Henceforth, we believe that such a dose response effect adds to the validity of downstream effects of the *COL6A3* variant.

Our previous work on *Col6a1−/−* mice reported an increased progression of OA pathophysiology, secondary to impaired PCM function. The latter is reflected by reduced PCM stiffness, increased cell swelling, and altered calcium response to osmotic stress.^[^ ^5^, ^8^ ^]^ Following the altered mechano‐transduction associated with aberrant COLVI function, we here focused particularly on changes in the cellular phenotype of chondrocytes governed by the damaging *COL6A3* R1504W variant in interaction with hyper‐physiological mechanical cues via transcriptome‐wide (co)‐expression and biological pathway analyses.

By combining and jointly analyzing hiPSC derived chondrocytes in two models namely embedded in cylindrical disc‐shaped agarose organoids^[^ ^35^ ^]^ and spherical neocartilage pellets^[^ ^13^, ^25^ ^]^ we aimed to obtain most consistent and robust results while taking advantage of the specific aspects of either model. The advantage of the first model is that it ensured equal distribution of mechanical stress throughout the disc‐shaped sample. The advantage of the second model is that it allows harnessing the original matrix deposited by the hiPSC‐derived chondrocytes thereby permitting the characterization of changes in the ECM. Despite the fact that we addressed reproducibility by combining and jointly analyzing data of two cartilage organoid models that were performed in two different laboratories with multiple differentiations, a weakness of our study is that the *COL6A3* R1504W mutation was introduced in only one hiPSC line. However, in silico off‐target analysis showed only five potential, but highly unlikely, off target sites. Moreover, Sanger sequencing confirmed that these were not affected by the CRISPR/Cas9 gene editing.

By isolating mutated COLVI protein from the neocartilage organoids we could show that it had reduced binding to fibronectin. Based on this finding it is tempting to hypothesize that this reduced binding is underlying the observed lack of the inflammatory response to hyperphysiological loading. In contrast to previous findings,^[^ ^11^ ^]^ we could not detect any binding of COLVI with hyaluronan, which might be explained by the source of the COLVI proteins, as they were extracted from the culture medium. Alternatively, other intermediary proteins are necessary for binding COLVI to hyaluronan which might not have been present in the medium.

We have used supervised machine learning with a recently developed convolutional neural network to quantify the differences in both sGAG size and numbers between isogenic controls and *COL6A3* variants.^[^ ^26^, ^38^ ^]^ This procedure relied on initial manual annotation of sGAG structures for supervised classification because the staining protocols used for TEM (a protocol including osmium tetroxide and potassium ferrocyanide, uranyl acetate, and lead citrate) are rather nonspecific and stain almost any cellular structure. Nonetheless, given that our annotated sGAG structures are very similar to previously annotated sGAGs in rat and mouse tissue,^[^ ^39^, ^40^ ^]^ our TEM results are in line with the results of the DMMB assay, and the Alcian blue staining, we are confident that the applied supervised machine learning approach has reliably annotated sGAG structures. Nonetheless, more specific staining that enhances contrast, e.g., carbon double bonds (Osmium) or charged structures (Ruthenium salts) could have reduced our initial manual annotation effort and increased sensitivity. Together, our data implies that the reduced sGAG found with the *COL6A3* variant is likely to be explained by a chondrocyte phenotype marked by increased expression of MMPs and reduced expression of *ACAN*. Next to a lacking initial upregulation of inflammatory signaling after mechanical loading, the aberrant phenotype of *COL6A3* chondrocytes persisted also 4 days after the hyper‐physiological mechanical loading regime. We can, however, not exclude the involvement of other collagen type VI‐mediated changes in the PCM function that could result in sGAGs reduction. Notable in this respect is the recently reported COLVI‐mediated role of Decorin in aggrecan retention to the PCM.^[^ ^41^ ^]^

A limitation of the current study is that we have initially relied on the predicted damaging effects of the identified R1504W variant on COLVI protein function by in silico tools (SIFT, Polyphen, Provean, i‐mutant V2.0, MUpro, and PANTHER‐PSEP) for prioritization on the damaging effect on protein function. Because the damaging effect was predicted with high confidence, we studied the effect of the variant on the chondrocyte phenotypic state using human in vitro cartilage organoid models. Due to ethical constraints, we did not address the clinical genotype‐phenotype relationship of carriers as this requires an in‐depth study of the (extended) pedigree, penetrance, age of onset, and focused phenotyping.

On a different note, applying human in vitro cartilage organoid models has refrained us from studying the effect of the R1504W *COL6A3* variant more physiological loading regimes, that could exert additional effects on the genotype–phenotype relationship. The latter because aberrant responses to physiological cues are likely subtle, that would require repetitive and long‐term loading. This was outside the scope of our study, as we aimed to investigate the short‐ and long‐term effects of acute, and injurious hyper‐physiological loading as outlined by previously^[^ ^42^ ^]^ and as marked by induction of proinflammatory mediators. Such acute injurious loading is generally accepted as a major cause for onset of post‐traumatic OA. Additionally, long‐term cultures of our cartilage organoids could evoke confounding factors such as dedifferentiation of the chondrocyte phenotype, or cell death that would refrain robust data generation. An in vivo mouse model would likely be eligible to study the effect of such physiological loading regimes and that is currently under investigation.

In characterizing the chondrocyte phenotype associated with the R1504W *COL6A3* variant, we have determined the overlapping transcriptome‐wide profile with that of OA chondrocytes. We must note that, while significant in implication, this overlap was only limited. This is likely explained by the fact that the transcriptome‐wide differences between lesioned and preserved OA cartilage particularly describe the overall OA‐associated chondrocyte phenotype in the general OA population which is likely different from the chondrocyte phenotype due to a specific variant in *COL6A3*. Nonetheless, we would like to argue that the significance of the DEG overlap between the *COL6A3* variant, and OA lies in the underlying pathways and biological functions of the genes involved such as ECM components (*COL27A1*, *COL11A2)*, and ossification (*SPP1*, *MGP*) as they describe critical aspects of the OA chondrocyte phenotype.

Together, using genetic engineering of hiPSC‐derived neocartilage organoid models while implementing hyper‐physiological mechanical loading conditions, we established a tailored model to study the biological function of proteins in the transduction of mechanical cues from ECM to chondrocytes, while complying with the societal wish to reduce animal models. By using this model, we showed that the COLVI protein variant had reduced binding to fibronectin, and we advocated that the observed lack of the initial inflammatory response to hyper‐physiological loading is likely secondary to this aberrant function of COLVI in the PCM. Additionally, we demonstrated that the initial inflammatory response to hyper‐physiological loading is particularly epigenetically regulated by the lncRNA *MIR31HG*.

## 4 Experimental Section

### Exome Sequencing

Exome sequencing of a patient with generalized OA at multiple joint sites was performed by Illumina HiSeq 2000 technology (Beijing Genome Institute) using the protocol described in the Supporting Information.

### Patient and Public Involvement

The patient participation OA Leiden group was consented during regular meetings on the setting and outcome measures of the research question. They were central to dissemination of the research.

### Ethics Approval

Any necessary ethics approval for the GARP study was secured by the committee medical ethics (CME) of the Leiden University Medical Centre reference number P76/98.

### hiPSC Line and Cell Culture

An hiPSC line as described earlier was used as the unedited isogenic control.^[^ ^24^ ^]^ In short, RVR‐iPSC line was retrovirally reprogrammed from BJ fibroblasts. Cells were characterized and their pluripotency (to three germ layers) was confirmed previously.^[^ ^24^, ^43^ ^]^ Further culture conditions can be found in the Supporting Information.

### Genome Editing of hiPSCs

CRISPR‐Cas9 single‐stranded oligonucleotide‐mediated homology‐directed repair in hiPSCs was employed to attain biallelic modification of rs144223596 (c.4510C>T) in an isogenic background. Additional information on the genomic target, guide sequences, and transfection protocol can be found in the Supporting Information.

### hiPSC Differentiation to Induced Chondrocytes

Generation of induced chondroprogenitor cells (hiCPCs) was based on a protocol previously described^[^ ^24^ ^]^ which was shown to produce similar neocartilage to that produced by human primary articular chondrocytes.^[^ ^44^ ^]^ In short: First, hiPSCs were progressed through the anterior primitive streak on day 0, paraxial mesoderm on day 1, early somite on day 2, sclerotome on days 3 to 5, and chondroprogenitor on days 6 to 14. Subsequently, these were washed with MD medium, dissociated with Gentle Cell dissociation medium (Stem Cell), and centrifuged for 5 min at 1200 rpm. Cell aggregates were subsequently maintained in chondrogenic differentiation (CD) medium. A more detailed description of the hiPSC differentiation process and creation of neocartilage organoids in described in the Supporting Information.

### Mechanical Loading

The spherical‐shaped neocartilage constructs were mechanically loaded using a MACH‐1 mechanical testing device (Biomomentum), at a rate of 5hz with 20% sinusoidal peak‐to‐peak strain for 10 min, as described earlier.^[^ ^25^ ^]^

### sGAG Measurement

Sulfated glycosaminoglycan (sGAG) concentrations in the neocartilage organoids (µg sGAG/µg DNA) were measured using the Farndale Dimethyl Methylene Blue (DMMB, Sigma) method.^[^ ^45^ ^]^ Additional information can be found in the Supporting Information.

### Histology and Immunohistochemistry

Neocartilage samples were fixed in 4% formaldehyde and embedded in paraffin. Sections were stained with Alcian Blue (Sigma‐Aldrich) and Nuclear Fast Red (Sigma‐Aldrich). Deposition of collagen II and collagen VI in the neocartilage constructs was visualized immunohistochemically according to the protocol described in the Supporting Information.

### RT‐qPCR

Per sample, two replicate mRNA samples were measured in triplicates in a MicroAmp Optical 384‐Well Reaction Plate (ThermoFisher Scientific), using the QuantStudio Flex Real‐Time PCR system (Applied Biosystems). Additional information can be found in the Supporting Information.

### RNAseq

RNA from neocartilage constructs was extracted 12 h postmechanical loading and analyzed using the Illumina NOVAseq 6000. Additional information on RNA isolation, mapping, alignment, and data processing and analysis can be found in the Supporting Information.

### Solid‐Phase Binding Assay

Conditioned medium of wild‐type and *COL6A3* variant organoids was collected and concentrated in preparation for the binding assay. To this end, 450 µl of medium was collected in 100 K molecular weight cutoff Pierce Protein Concentrators (Thermo Scientific) and centrifuged for 10 min at 12000 *g*. Subsequently, COL6 concentration was determined using the Human *COL6A3* ELISA Kit (Assay Genie) according to the manufacturer's protocol which can be found in the Supporting Information.

### Transmission Electron Microscopy

For the neocartilage organoids, consisting of cells and matrix, a previously established protocol was used to perform TEM. Additional information on sample processing, image acquisition, processing and analysis can be found in the Supporting Information.

### Validation of Regulatory Effects MIR31HG on Protein‐Coding Gene Expression

Primary chondrocytes were isolated from 12 independent donors and passaged twice, as previously described.^[^ ^46^ ^]^ Chondrocytes were transfected in duplo with LNA GapmeR (Qiagen) targeting *MIR31HG* (AGTGCAGCAAAATTAG) or GapmeR negative control (AACACGTCTATACGC) at 10 × 10^−9^ m final concentration using Lipofectamine RNAiMax Transfection Reagent according to instructions of the manufacturer (Invitrogen). Cells were lysed 24 h posttransfection and RT‐qPCR was performed as described above for *GAPDH, SDHA*, *MIR31HG, PTGS2, IRAK2, PTGER4, IL1R1*. Relative gene expression levels were calculated with the 2‐ΔΔCt method, using *GAPDH* and *SDHA* as internal control. A t‐test was performed on the −ΔCt values, and P values less than 0.05 were considered significant.

### Statistical Analysis

For differential expression analysis, a negative binomial generalized linear model using the R DESeq2 package was used. Please find the Supporting Information for the description in full of our RNAseq data processing and differential gene expression analysis. For all other data analyses, a generalized linear model was used including the factors hyper‐physiological loading and the *COL6A3* variant using R statistical software version 4.1.1. A two‐sided test with a *P*‐value of 0.05 as a significance cutoff was used. The alignment of the data with the presumptions of the generalized linear model was tested both visually using boxplots/QQ‐plots, as well as formally using the Kolmogorov‐Smirnov test. The reported beta value represents the standardized coefficient, indicating the change in the dependent variable's standard deviations for each standard deviation change in the predictor variable. Statistics in figure legends are reported as beta ± standard error.

## Conflict of Interest

The authors declare no conflict of interest.

## Author Contributions

F.G. and I.M. contributed equally to this work. N.G.C.B., Z.H., A.R.D., Y.F.M.R., F.G., and I.M. developed the concept of this study; N.G.C.B., Z.H., G.M., S.S.A., A.R.D., G.H., N.S., M.K., R.I.K., A.A.M., B.B.R.K., and F.G. acquired materials and data; N.G.C.B., R.C.D.A., B.B.R.K., Y.F.M.R., F.G., and I.M. analyzed the data; and all authors contributed to the writing of the manuscript.

## Supporting information

Supporting Information

Supporting Tables

## Data Availability

The data that support the findings of this study are available in the Supporting Information of this article.
